# Complete Genome Sequence of Stutzerimonas stutzeri Strain SOCE 002, a Marine Bacterium Isolated from the Surface Seawater of Dapeng Bay

**DOI:** 10.1128/mra.00150-23

**Published:** 2023-04-17

**Authors:** Jing Guo, Shuaishuai Xu, Yanting Liu, Chuanlun Zhang, Shengwei Hou

**Affiliations:** a Department of Ocean Science and Engineering, Southern University of Science and Technology, Shenzhen, China; b Southern Marine Science and Engineering Guangdong Laboratory, Guangzhou, China; c Shenzhen Key Laboratory of Marine Archaea Geo-Omics, Southern University of Science and Technology, Shenzhen, China; d College of Life Science and Technology, Jinan University, Guangzhou, China; Portland State University

## Abstract

We report the complete genome sequence of Stutzerimonas stutzeri strain SOCE 002, obtained from Illumina and Oxford Nanopore sequencing. The genome is 4.68 Mb long, with a GC content of 63.5%, and contains 4,334 protein-coding genes, 60 tRNAs, and 12 rRNAs. We expect that this complete genome sequence will provide a reference for both genomic and metabolic analyses of S. stutzeri.

## ANNOUNCEMENT

The genus *Stutzerimonas* had previously been ascribed to the Pseudomonas genus as Pseudomonas stutzeri, within the *Pseudomonadaceae* family (1). Stutzerimonas stutzeri strains are widely distributed in terrestrial and marine environments (2) and are ecologically important due to their versatile metabolic capabilities, including denitrification (3, 4), nitrogen fixation (5–7), and degradation of aromatic hydrocarbons (8). Here, we present the complete genome sequence of S. stutzeri strain SOCE 002, a marine strain that was isolated from seawater collected at XIASHA (22°52′43″N, 114°01′30″E) in Dapeng Bay, China.

The measured salinity, temperature, and chlorophyll *a* concentration of the surface seawater were about 30 ppt, 24°C, and 5.8 μg/L, respectively. One liter of seawater was filtered through a 0.22-μm nitrocellulose membrane and placed aseptically on Marine Broth 2216 agar (BD Difco, Franklin Lakes, NJ, USA). Bacterial colonies were isolated by the streak plating method, and selected isolates were grown in Marine Broth 2216 liquid medium for 48 h at 28°C. The genomic DNA was extracted from cell pellets by the cetyltrimethylammonium bromide (CTAB) method (9) and quantified using the Qubit double-stranded DNA broad-range kit (Thermo Fisher Scientific, USA). Sequencing was performed at Novogene Bioinformatics Technology Co., Ltd. (Tianjin, China). For Illumina sequencing, 200 ng DNA was sheared into ~350-bp-long fragments using the LE220R-plus ultrasonicator (Covaris, Woburn, MA, USA), and then DNA fragments were end repaired and poly(A) tailed for library preparation with the NEBNext Ultra DNA library preparation kit (New England Biolabs, Ipswich, MA, USA) following the manufacturer’s instructions. Illumina sequencing was performed on a NovaSeq 6000 platform (Illumina, San Diego, CA, USA) with the 2 × 150-bp paired-end strategy, and 67.523 Gbp of raw reads were generated. Readfq v8 (https://github.com/cjfields/readfq) was used to clean the Illumina raw reads for subsequent analysis, including removal of reads with a significant number (*n* = 40) of low-quality bases (default quality threshold value of ≤38), reads with excessive (*n* = 10) uncalled bases, and reads with a certain overlap (15 bp) with adapter sequences, which generated a total of 67.385 Gbp of clean reads.

For Nanopore sequencing, DNA fragments longer than 10 kb were selected with BluePippin (Sage Science, Beverly, MA, USA). Nanopore libraries were prepared with a ligation sequencing kit (SQK-LSK109; Oxford Nanopore Technologies, Oxford, UK) following the manufacturer’s recommendations and were sequenced on the PromethION 48 platform with a R9.4.1 flow cell. After base calling using Guppy v6.17 (Oxford Nanopore Technologies) (10), a total of 11.348 billion reads were obtained, with an average length of 2,502.63 bp and an *N*_50_ value of 4,096 bp. Nanopore quality control was achieved using NanoPlot v1.18.2 with a threshold Q value of >7, and low-quality regions were trimmed using NanoFilt v2.7.1 (11). The assembly process was then performed by Flye v2.4.2 in metagenomic mode (12), and the assembly was polished using Pilon v1.24 (13) and NextPolish v1.4.0 (14) with short reads generated from the Illumina platform. Unicycler v0.4.8 (15) and Bandage v0.8.1 (16) were used to identify and clean circular contigs. All tools were run with default parameters unless otherwise specified.

Gene prediction and annotation were performed using the NCBI Prokaryotic Genome Annotation Pipeline (PGAP) (17). The genome size was 4.68 Mb, with a GC content of 63.51%. There were 4,334 protein-coding genes, 4 stretches of 5S-16S-23S rRNA genes, and 60 tRNA genes. The 16S rRNA gene sequences of S. stutzeri strain SOCE 002 were found to be 99.64% identical to those of the P. stutzeri type strain ATCC 17588 (equivalent to CGMCC 1.1803, GenBank accession number CP002881), and the genomic average nucleotide identity (ANI) of the two strains was 98.4%. The complete genome sequence of S. stutzeri strain SOCE 002 will facilitate understanding of its mechanisms for survival and proliferation in the human-impacted seawater of Dapeng Bay.

### Data availability.

The complete genome sequence of S. stutzeri strain SOCE 002 has been deposited in GenBank under the BioProject accession number PRJNA895221, the BioSample accession number SAMN31497883, and the GenBank accession number CP113890. The SRA accession numbers of the Illumina and Nanopore reads are SRR22365957 and SRR22365958, respectively.
